# Isavuconazole as primary antifungal prophylaxis in pediatric hematological patients: a clinical and pharmacokinetic analysis

**DOI:** 10.1128/aac.00451-26

**Published:** 2026-06-26

**Authors:** Francesco Baccelli, Caterina Campoli, Martina Colli, Milo Gatti, Francesca Gottardi, Gennaro Pagano, Francesco Venturelli, Davide Leardini, Edoardo Muratore, Gianluca Bossù, Federico Pea, Pierluigi Viale, Maddalena Giannella, Tamara Belotti, Arcangelo Prete, Riccardo Masetti

**Affiliations:** 1 Pediatric Hematology and Oncology, IRCCS Azienda Ospedaliero-Universitaria di Bologna, Bologna, Italy; 2 Department of Medical and Surgical Sciences, University of Bologna 9296, Bologna, Italy; 3 Infectious Diseases Unit, IRCCS Azienda Ospedaliero-Universitaria di Bologna, Bologna, Italy; 4 Clinical Pharmacology Unit, Department for Integrated Infectious Risk Management, IRCCS Azienda Ospedaliero-Universitaria Di Bologna, Bologna, Italy; 5 Department of Pediatric Hematology-Oncology, Azienda Unità Sanitaria Locale di Rimini, Rimini, Italy; Queen Mary University of London, London, United Kingdom

**Keywords:** isavuconazole, antifungal prophylaxis, invasive fungal disease, pediatric, hematology, oncology, cancer, hematopoietic stem cell transplantation, therapeutic drug monitoring

## Abstract

Invasive fungal diseases (IFDs) are a major threat in pediatric patients with hematologic malignancies (HMs) and hematopoietic stem cell transplantation (HSCT) recipients. Optimal antifungal prophylaxis (AFP) remains a clinical challenge. Isavuconazole (ISA), a broad-spectrum triazole, may offer a safer, more predictable option for AFP in this setting, but pediatric data are scarce. We retrospectively analyzed pediatric patients (≤18 years) with HM or HSCT who received isavuconazole as primary AFP from January 2021 to April 2025 at a single center. Isavuconazole dosing, therapeutic drug monitoring (TDM), safety, and breakthrough IFD incidence were systematically evaluated in a standardized stewardship framework. Isavuconazole was administered as primary antifungal prophylaxis in 21 patients (median age 9.6 years), 17 (77.3 %) after HSCT. Doses were weight-adjusted (100 or 200 mg/day) with loading over 48 h. No severe adverse events attributable to isavuconazole were reported. No breakthrough IFDs occurred during prophylaxis. Concomitant use of CYP3A4-interacting agents (e.g., ruxolitinib) was not associated with increased toxicity or isavuconazole level alterations. TDM was performed in all patients (188 total measurements); median Cmin was 3.4 mg/L. The target range (1–5 mg/L) was achieved in 95.2% of first TDMs. Dosage adjustments were needed in only 5.9% of cases, indicating consistent pharmacokinetics. In this retrospective cohort of high-risk pediatric hematology patients, isavuconazole used as primary antifungal prophylaxis showed favorable safety, consistent drug exposure, and no breakthrough IFDs. These findings support further prospective evaluation of isavuconazole in pediatric prophylactic strategies.

## INTRODUCTION

Invasive fungal disease (IFD) represents a potential cause of morbidity and mortality among pediatric patients with hematologic malignancies (HM) and those undergoing allogeneic hematopoietic stem cell transplantation (HSCT) (1). Antifungal prophylaxis (AFP) represents a cornerstone in the supportive care of these patients. The latest pediatric-specific recommendations by European Conference on Infections in Leukemia (ECIL) highlight the need for an adequate primary AFP in patients who have the probability ≥10% of IFD, including high-risk acute lymphoblastic leukemia (HR-ALL), relapsed ALL (ALL-Rel), acute myeloid leukemia (AML), and allogeneic HSCT recipients, particularly those affected by graft-versus-host disease (GVHD) (2). ECIL-10 2025 update confirmed the need for AFP in high-risk pediatric patients, including those ≥12 years old, and those with ALL who did not completely respond to induction (3, 4).

The choice of the best antifungal agent used for prophylaxis is a matter of debate in children as it should balance several key principles: safety and tolerability, pharmacokinetics with reliable predictability, ecological impact, and cost-effectiveness (5). Azoles, particularly voriconazole and posaconazole, are commonly used in this setting, but both are associated with significant limitations due to pharmacokinetic variability, significant drug interactions, and safety concerns (6). Voriconazole, despite its broad-spectrum activity, exhibits highly variable pharmacokinetics in children, necessitating frequent therapeutic drug monitoring (TDM) and dosage adjustments to avoid subtherapeutic levels or toxicity. Posaconazole, though effective, has unpredictable bioavailability in younger children and a high potential risk of interactions via cytochrome P450/3A4 (CYP3A4) inhibition (1, 6).

Other antifungal molecules can be used among pediatric patients, despite even ECIL guidelines define them as second choice (2, 4). Among them, liposomal amphotericin B remains highly effective, with limited drug-drug interaction and reduced costs due to generic availability (7); however, it requires intravenous administration and constant monitoring of renal and hepatic function, especially among children undergoing intensive chemotherapy regimens or HSCT (8, 9).

Moreover, recent epidemiological reports have described a shift toward Aspergillus species with intrinsic or reduced susceptibility to amphotericin B (e.g., *A. terreus*, *A. flavus*) (10). Echinocandins offer an excellent safety profile but require daily intravenous administration, and they have demonstrated a clear fungicidal effect against only yeasts (7). Isavuconazole (ISA) is a newer, broad-spectrum second-generation triazole approved by EMA and FDA since 2024 for both pediatric and adult populations as treatment of invasive aspergillosis and mucormycosis (11, 12). Approval was supported by two prospective pediatric studies. A phase 1 trial assessed the safety, tolerability, and population pharmacokinetics of intravenous and oral isavuconazonium sulfate in immunocompromised pediatric patients aged 1 to <18 years, showing that the pediatric dosing strategy achieved exposures broadly comparable to those observed in adults and was generally well tolerated (13). In a phase 2 trial, isavuconazole resulted safe and effective for the treatment of IFD in children with IFD (13). Nowadays, isavuconazole is not licensed for prophylaxis in either adults or children, and its use as primary AFP remains off-label. In the largest multicenter pediatric real-world cohort published to date, 107 children and adolescents received isavuconazole, supporting its favorable safety profile and potential role in pediatric invasive fungal disease management, with TDM providing additional exposure guidance. Only 12 patients in this cohort received isavuconazole as AFP (14).

Isavuconazole is given as a prodrug (isavuconazonium sulfate) that exhibits high oral bioavailability, reaching approximately 98% in adults. Following rapid and complete absorption, the prodrug is swiftly and fully cleaved to its active molecule, isavuconazole. Oral and intravenous formulations are interchangeable, as food intake and gastric pH variations do not affect isavuconazole absorption, thus in contrast with posaconazole (7, 15, 16). It offers several theoretical advantages over other azoles, including linear pharmacokinetics, excellent oral bioavailability (>97%), predictable serum levels, a favorable safety profile, and a lower risk of drug interactions due to mild CYP3A4/A5 modulation with minor additional biotransformation mediated by uridine diphosphate-glucuronosyltransferase (UGT) pathways. In fact, isavuconazole shows extensive tissue distribution, including the liver, lungs, eyes, kidneys, skin, bone, nasal mucosa, and brain (5). In the pediatric setting, an additional advantage is the availability of an intravenous formulation for patients unable to take oral medications (1). Unlike voriconazole, TDM is generally not required for isavuconazole in adults, while pharmacokinetic data in children remain limited (13, 15, 17–23). A recent systematic review supported the potential role of TDM-guided strategy in case of prophylaxis/treatment among pediatric patients (24).

However, at this time, no study systematically evaluated pharmacokinetics in parallel with clinical outcomes of isavuconazole used specifically as primary prophylaxis in children. The present study aims to assess the feasibility, pharmacokinetic profiles, and efficacy of isavuconazole when used as primary AFP in pediatric patients with HM or undergoing HSCT.

## MATERIALS AND METHODS

We conducted a retrospective, monocentric observational study among patients ≤18 years old with HM or undergoing HSCT who received isavuconazole as primary AFP from January 2021 to April 2025 in the Pediatric Hematology and Oncology of the IRCCS Azienda Ospedaliero-Universitaria di Bologna, in Italy. This group of patients received isavuconazole as an off-label treatment, after the approval by the local ethics committee (Comitato Etico Area Vasta Emilia Centro) and after receiving informed consent form signed by parents or legal guardians. Isavuconazole was used as primary AFP from January 2021 on a single-patient basis, within a multidisciplinary antifungal stewardship program involving pediatric hematology-oncology, infectious diseases, and clinical pharmacology specialists. A real-time TDM-guided strategy for optimizing dosing regimen was adopted, as previously reported (1). Isavuconazole was preferentially adopted in patients requiring prolonged mold-active prophylaxis, in case of concomitant administration of drugs metabolized through cytochrome P450 (CYP3A4) (e.g., ruxolitinib, vincristine, or venetoclax), previous intolerance or contraindication to other azoles, or nephrotoxicity concerns associated with liposomal B amphotericin. Duration of prophylaxis was also individualized according to the persistence of patient’s fungal risk.

Initial dosing was decided according to pediatric pharmacokinetic recommendations published (1, 25, 26). In patients <30 kg, they received 100 mg/day, whereas patients ≥30 kg, 200 mg/day. A tripled loading dose was administered for the first 48 h to reach steady-state plasma levels. TDM was first performed on day +3 from loading dose, and subsequently every 7–14 days, or earlier in case of clinical instability, gastrointestinal absorption issues, concomitant inflammatory events, or changes in co-treatment. All dosage regimens were reported as isavuconazole active moiety equivalents. Therefore, the corresponding amount of the prodrug isavuconazonium sulfate would be higher: specifically, 200 mg of isavuconazole corresponds to 372 mg of isavuconazonium sulfate, and 100 mg of isavuconazole corresponds to approximately 186 mg of isavuconazonium sulfate.

Isavuconazole was administered orally as whole capsules (100 mg) in children able to swallow. Patients unable to take oral medications due to swallow difficulties (e.g., mucositis in the immediate post-HSCT phase) received the intravenous or galenic formulation with the same dosage. The galenic oral formulation was compounded according to a standardized local hospital procedure. The manipulation of the commercially available formulation was performed to obtain the closest feasible approximation of the prescribed daily dose.

Plasma mean trough concentration (Cmin) of isavuconazole was determined via liquid chromatography coupled with tandem mass spectrometry (LC-MS/MS) in the Clinical Pharmacology Unit laboratory. The desired isavuconazole Cmin was set between 1.0 and 5.0 mg/L, based on pharmacokinetic-pharmacodynamic (PK/PD) targets for prophylaxis and consensus expert guidance (1, 7, 25).

Clinical parameters collected for all patients included duration of prophylaxis, liver and kidney function laboratory and QTc interval monitoring, onset of adverse events (AEs) with grading according to CTCAE v5.0, concomitant treatments, and occurrence of breakthrough IFD during isavuconazole prophylaxis. Breakthrough IFD was defined, according to EORTC/MSGERC 2020 criteria, as any proven or probable IFD that occurred after ≥5 days of isavuconazole prophylaxis and within 7 days of discontinuation, in the absence of therapeutic switch (27).

All patients on isavuconazole prophylaxis underwent serial serum galactomannan surveillance during neutropenia or the early post-HSCT. Chest CT scan was performed in case of persistent fever >96 h despite broad-spectrum antibiotics, and bronchoalveolar lavage with microbiological investigations and fungal biomarkers was carried out in patients with radiological suspicion of IFD, in accordance with ECIL recommendations (4).

According to our institutional protocol for febrile neutropenia, patients receiving mold-active prophylaxis did not systematically undergo empirical antifungal treatment at the onset of fever. During febrile/aplastic neutropenia, isavuconazole prophylaxis was continued, and specific fungal diagnostic reassessment was performed only in case of persistent/recurrent fever, clinical deterioration, suggestive radiological findings, positive fungal biomarkers, or microbiological evidence of invasive fungal disease. A switch to systemic antifungal treatment was reserved for patients with suspected or documented breakthrough invasive fungal disease (bIFD).

## RESULTS

### Study population

Twenty-one pediatric patients who received isavuconazole as AFP were included in this study. The median age at the start of isavuconazole prophylaxis was 9.6 years (range 3.0–18.0 years). The median weight of patients was 34.3 kg (range 11.6–78.0 kg). In Table 1, the main characteristics of patients are summarized. The most frequent diagnoses were ALL (42.8%) and AML (23.7%). Seventeen (77.3%) patients received isavuconazole prophylaxis after allogenic HSCT, 3 (17.6%) starting concomitantly with the conditioning regimen, 6 (35.3%) in the immediate post-HSCT phase (<30 days after CSE infusion), and 8 (47%) later than 30 days after HSCT with a mean time after of 5.6 months (3–11). Patients received isavuconazole as prophylaxis in the context of ongoing GVHD that necessitated treatment with steroids and/or Ruxolitinib or in case of delayed/poor immune reconstitution following HSCT. Of the 17 HSCT recipients, 13 (76.5%) were transplanted in first complete remission, while 4 (23.5%) in second or subsequent remission. One patient was classified in both the CHT and HSCT groups, given that isavuconazole prophylaxis was used during both phases of treatment. In Table 2, the main characteristics of HSCT patients are summarized.

**TABLE 1:** Main demographic and clinical characteristics of the study patients; *n*, number^*a*^

	*N* = 21
Age, years, median (range)	9.6 (3.0–18.0)
Age classes, n (%)	
1 to <7 yr	7 (33%)
7 to <12 yr	10 (47.6%)
12 to <18 yr	4 (19%)
Sex, male – female, *n* (%)	15 (71.4%) – 6 (28.6%)
Weight, kg, median (range)	30 (11.6–78)
Weight classes, *n* (%)	
<30 kg	11 (52.4%)
≥30 kg	10 (47.6%)
Underlying disease, n (%)	
ALL	
- First diagnosis	5 (23.8%)
- Relapse	4 (19%)
AML	
- First diagnosis	4 (19%)
- Relapse	1 (4.7%)
SAA	2 (9.5%)
Lymphoma (T-lymphoblastic lymphoma/anaplastic large cell lymphoma)	2 (9.5%)
JMML	2 (9.5%)
LAD-1	1 (4.7%)
AFP indication, *n* (%)	
HM on intensive chemotherapy	5 (22.7%)
HSCT	17 (77.3%)

^*a*^ HM, hematological malignancies; HSCT, hematopoietic stem cell transplantation; ALL, acute lymphoblastic leukemia; AML, acute myeloid leukemia; SAA, severe aplastic anemia; JMML, juvenile myelomonocytic leukemia; LAD-1, leukocyte adhesion deficiency 1.

**TABLE 2:** Main characteristics of HSCT population^*a*^

	*N* = 21
ALLO-HSCT	17.0 (100%)
Duringconditioning regimenn	3 (17.6%)
Acute post-HSCT (<30 days)	6.0 (35.3%)
Post-HSCT (>30 days)	8.0 (41.7%)
Conditioning regimen	
MAC	15 (88.2%)
RIC	2.0 (11.8%)
Donor	
MUD	7.0 (41.8%)
Sibling	2.0 (11.8%)
HAPLO	8.0 (47.0%)
Source of CSE	
BM	10.0 (58.8%)
PBSC	7.0 (41.2%)

^*a*^ HSCT, hematopoietic cell transplantation; MAC, myeloablative conditioning; RIC, reduced-intensity conditioning; MUD, matched-unrelated donor; HAPLO, Haploidentical; BM, bone-marrow; PBSC, peripheral-blood stem cells.

### Isavuconazole administration

The initial administered continuation dose was 100 mg/day in 16 patients weighing <30 kg, whereas five patients weighing ≥30 kg received 200 mg/day. Drug formulations included oral capsules in 16 patients and galenic formulation in 5, respectively. All patients that originally started with the galenic formulation were able to switch to the conventional oral one during the treatment period. One patient received initially isavuconazole IV, shifting to oral formulation with clinical condition improvement. The median duration of isavuconazole treatment was 5 months (range: 2–14) for patients who have undergone HSCT and 5 months (range: 1–8) for those receiving chemotherapy.

### Adverse events, drug interactions, and breakthrough IFD

No severe AEs attributable to isavuconazole were observed during the study period. One patient precautionarily discontinued isavuconazole due to a grade two transaminase increase. The patient experienced a severe, multisystemic chronic GVHD involving the liver. No significant changes in transaminase values were observed after isavuconazole withdrawal, further supporting the absence of a direct causal relationship, allowing the reintroduction of isavuconazole. Moreover, TDM levels of isavuconazole were always in range. No clinically relevant pharmacological interactions were detected. Specifically, in patients receiving concomitant treatment with CYP3A4-modulated drugs with potential toxicity due to plasmatic overexposure, no alterations in isavuconazole trough levels were observed, nor were any increased toxicities of the co-administered drugs (e.g., cytopenia in ruxolitinib-treated patients) reported. Among the four patients who received isavuconazole during conditioning regimen, no significant drug interactions were reported by the treating physician. TDM confirmed target busulfan exposure throughout conditioning in these patients. All patients, with the exception of the three undergoing conditioning regimen for HSCT, were on trimethoprim-sulfamethoxazole prophylaxis. It is important to underline that among patients undergoing chemotherapy protocols, two received concomitant treatment with vincristine, without any further AEs Table 3 . No case of breakthrough IFD on isavuconazole prophylaxis was reported during the observational study period. The median follow-up was 23 months (IQR, 15–39). Four patients died during the observation period due to causes unrelated to invasive fungal disease. No cases of breakthrough IFD were documented throughout follow-up, even after isavuconazole discontinuation.

**TABLE 3:** Isavuconazole co-administration with other therapies^*a*^

Drug class	Main agents	Patients, *n* (%)
Immunosuppressive/GvHD-directed therapy		
Calcineurin inhibitors	Cyclosporine, tacrolimus	18 (85.7%)
Mycophenolate mofetil	Mycophenolate mofetil	12 (57.1%)
Systemic corticosteroids	Prednisone, methylprednisolone	11 (52.4%)
JAK inhibitors	Ruxolitinib	13 (61.9%)
T-cell co-stimulation inhibitors	Abatacept	6 (28.6%)
Other immunomodulators	Belumosudil	1 (4.8%)
Antiviral therapy		
Anti-HSV/VZV	Acyclovir	18 (85.7%)
Anti-CMV (prophylaxis)	Letermovir	11 (52.4%)
Anti-CMV (treatment)	Valganciclovir	5 (23.8%)
Anti-*Pneumocystis jirovecii* prophylaxis	Trimethoprim–sulfamethoxazole	19 (90.5%)
Concomitant antineoplastic therapies		
Conventional chemotherapy	AIEOP protocols (IntreALL SR/HR)	2/1 (14.3%)
Vincristine	Vincristine	2 (9.5%)
Azacitidine		2 (9.5%)
Ponatinib		1 (4.3%)
Venetoclax		3 (14.3%)
Supportive medications		
Antiepileptics	Clobazam, gabapentin, levetiracetam	6 (28.6%)
Antihypertensives	Amlodipine, bisoprolol, doxazosin, enalapril	8 (38.1%)
Gastroprotective agents	Lansoprazole, pantoprazole	11 (52.4%)
Other	Ursodeoxycholic acid, FEC, anxiolytics	4 (19.0%)

^*a*^ GvHD, graft versus host disease; HSV, herpes virus; VZV, varicella zoster virus; CMV, cytomegalovirus; FEC, extracorporeal photo-apheresis.

### Therapeutic drug monitoring

**Fig 1:**
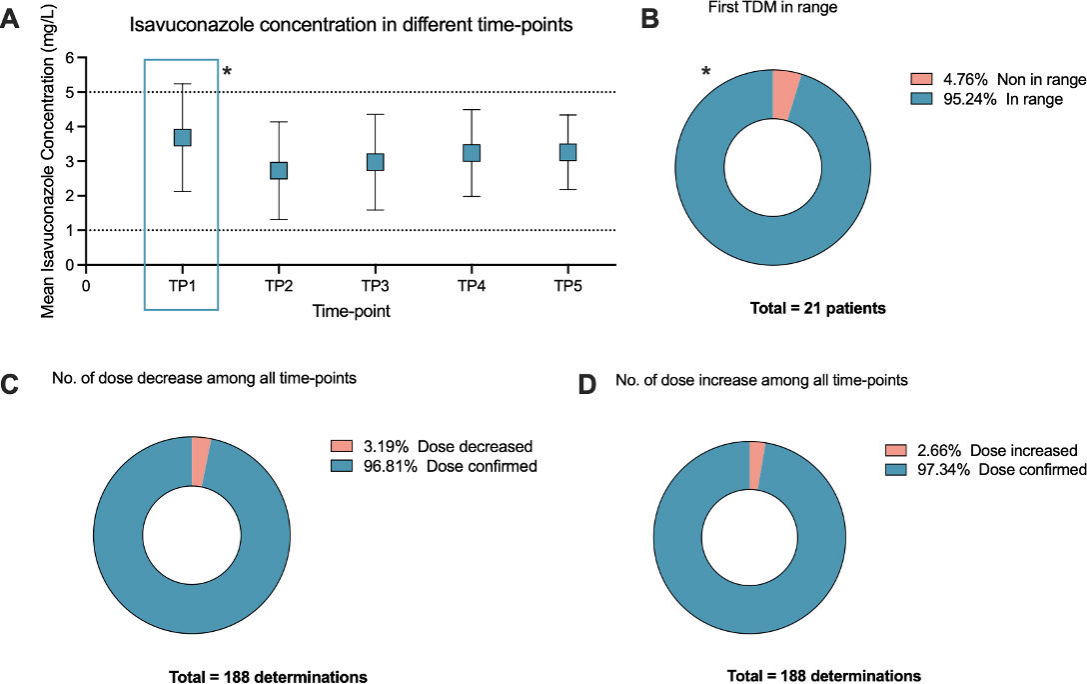
TDM-guided stability of isavuconazole exposure, TP1 represents the first isavuconazole trough concentration obtained after completion of the loading dose and initiation of standard maintenance dosing. TP2–TP5 represent subsequent weekly therapeutic drug monitoring assessments. (**A**) Mean isavuconazole trough concentrations at different therapeutic drug monitoring time-points. The dotted horizontal lines indicate the predefined therapeutic range of 1–5 mg/L. TP1 represents the first therapeutic drug monitoring assessment after treatment initiation, while TP2–TP5 represent subsequent monitoring time-points. (**B**) Proportion of patients with the first therapeutic drug monitoring assessment within the target range. (**C**) Proportion of isavuconazole determinations leading to dose decrease among all therapeutic drug monitoring assessments. (**D**) Proportion of isavuconazole determinations leading to dose increase among all therapeutic drug monitoring assessments.

A total of 188 TDM-guided expert clinical pharmacological advice (ECPA) was performed, with a median of 7 per patient (range, 2–28). The Cmin of isavuconazole was 3.4 mg/L (range 1.8–5.8 mg/L), falling within the recommended target range of 1–5 mg/L. The first TDM performed was in the therapeutic range for 20 of 21 patients (95.2%). In six cases (3.2%) (three measurements for the same patient, one each for the other three), a dose increase was required due to subtherapeutic Cmin; in five cases (2.7%) (one patient each), a dose decrease was required. In the five patients who initially received galenic formulation, one required dose adjustment due to subtherapeutic trough concentration. Overall, the dosage was confirmed in 177 of 188 measurements (94.1%). In Table 4 and Fig. 1, TDM data are summarized.

**TABLE 4:** Isavuconazole administration and therapeutic drug monitoring data^*b*^

	*N* = 21
Formulation	
Tablets	16 (76.2%)
Galenic formulation	5 (23.8%)
Prophylaxis duration, months, median (range)	
HSCT	6 (2–14)
Chemotherapy	5.2 (1–8)
Dose/kg/day (mg/kg) according to weight, median (range)	
<30 kg	4 (3.8–8.6)
≥30 kg	3.3 (1.8–5.7)
Cmin (mg/L) according to weight, median (range)	
<30 kg	2.5 (1.8–3.9)
≥30 kg	3.4 (2.1–5.8)
Number of determinations requiring dose increase, *n* (%)	5 (2.7%)
Number of determinations requiring dose decrease, *n* (%)	6 ^*a*^(3.2%)
Number of determinations with first TDM evaluation in range, *n* (%)	20 (95.2%)

^*a*^ One patient required three times a dose adjustment.

^*b*^ Cmin, mean trough concentration; TDM, therapeutic drug monitoring, HSTC: hematopoietic stem cell transplantation.

## DISCUSSION

We described a cohort of pediatric patients with onco-hematological diseases who received isavuconazole as primary prophylaxis for IFD. Longitudinal homogeneous TDM of the whole cohort was determined, providing paired clinical and pharmacokinetic data that are still lacking for the use as prophylaxis in pediatric setting. Isavuconazole was selected only after individualized multidisciplinary evaluation, mainly in patients requiring possible prolonged prophylaxis, receiving concomitant CYP3A4-metabolized therapies, presenting intolerance or contraindication to other azoles or liposomal B amphotericin. This patient-specific selection process should be considered when interpreting the favorable safety and pharmacokinetic profile observed in the present cohort.

In our cohort, isavuconazole was administered for a median duration of 5 months with a loading dose of 100 mg every 8 h for 2 days in case of children weighing <30 kg and 200 mg every 8 h for those above 30 kg (25); subsequently a standard daily dose of 100 mg/day in 60% of patients and 200 mg/day in 40%, corresponding to a median dose/kg/day of 4 mg/kg for children <30 kg and 3.3 mg/kg for those ≥30 kg. These doses are in line with previous pediatric reports and pharmacokinetic modeling suggesting that children require higher mg/kg dosing than adults to achieve comparable drug exposure due to increased drug clearance in this population (25, 28, 29). The relatively long median duration, 5 months of isavuconazole prophylaxis should be interpreted in light of the selected high-risk population included in this study. In many HSCT recipients, the risk of IFD extended beyond the early post-transplant neutropenic phase because of ongoing graft-versus-host disease, systemic immunosuppressive treatment, or delayed immune reconstitution. Therefore, isavuconazole was continued until resolution or substantial improvement of the underlying risk condition, rather than for a predefined fixed duration.

We reported an excellent safety and clinical efficacy profile of isavuconazole as AFP. Notably, no severe AEs were reported, corroborating findings from pediatric case reports and small series that define a reassuring safety level for isavuconazole (1, 21, 22, 25). This is clinically relevant because the study population included highly fragile patients, many of whom were exposed to multiple concomitant therapies with potential overlapping toxicities. No clinically relevant pharmacokinetic interactions were identified. Two patients received concomitant vincristine therapy without experiencing any adverse events. Although the sample size is limited and further prospective studies are warranted, this observation may highlight an additional potential advantage of isavuconazole compared with other azoles, which have traditionally been associated with clinically relevant vincristine-related toxicities (30, 31). Similarly, in 14 patients receiving ruxolitinib, no increased incidence of ruxolitinib-related toxicities (e.g., cytopenia) was reported, as described in case of concomitant administration of other azole (32). This favorable interaction profile is of particular interest in the evolving pediatric landscape, where novel therapies with potential overexposure in concomitance with azoles are increasingly used, such as venetoclax (32–34). One patient also underwent treatment with fecal microbiota infusion to treat chronic intestinal GVHD while he was on isavuconazole prophylaxis; no side effects were recorded (35).

The absence of breakthrough IFD further supports the clinical efficacy of isavuconazole AFP, even in high-risk populations such as early post-transplant recipients or those with ongoing GVHD. Importantly, despite the high expected incidence of febrile neutropenia in this population, no patient required empirical antifungal therapy or switch from isavuconazole to another antifungal agent in the absence of suspected or documented breakthrough invasive fungal disease. Our results are consistent with the available pediatric literature, while adult data reported higher rates of breakthrough IFDs in similar settings (26, 36).

Isavuconazole pharmacokinetics in pediatric patients differ from adults, with enhanced clearance, age-dependent metabolic rates, and variability in gastrointestinal absorption (especially in the context of mucositis or gastrointestinal toxicity), reinforcing the value of TDM (37, 38). In our cohort, isavuconazole exposure was stable across TDM monitoring. Out of 188 total TDM assessments, 177 (94.1%) resulted in confirmation of the initial dose, while only 11 measurements (5.9%) led to dose adjustment (six dose reductions, five increases). Importantly, the first TDM was already in range in 20/21 patients (95.2%). This high rate of target attainment without frequent adjustments suggests reliable pharmacokinetics of isavuconazole in prophylaxis settings. Previous studies (28, 29) showed the need for higher than standard doses, especially in severe infections but were based largely on treatment rather than prophylaxis setting. These findings suggest that during acute infections, factors such as inflammation, increased clearance, and altered metabolism necessitate dose adjustment, whereas in prophylactic settings these alterations are less pronounced. (39).

In our cohort, most patients achieved through concentrations within the predefined target range early after treatment initiation, and dose adjustments were rarely required. These findings suggest that the adopted pediatric dosing strategy provided stable exposure in the prophylactic setting. Therefore, our results do not demonstrate a clear need for intensive routine TDM in all pediatric patients receiving isavuconazole prophylaxis, particularly if low exposure thresholds (1 mg/L) for prophylactic efficacy will be validated in future studies. However, given the limited pediatric evidence, the off-label nature of prophylactic use, and the potential for pharmacokinetic variability in selected clinical conditions, TDM may still be useful as a confirmatory and safety tool in specific high-risk situations, such as unexpected toxicity, suspected drug–drug interactions, concerns about absorption, and non-standard formulations.

This study is limited by its retrospective design and the relatively small number, reducing the generalizability of these findings. Moreover, we did not perform TDM of co-administered drugs, limiting the ability to fully evaluate pharmacokinetic interactions. Our preliminary findings support the favorable safety and efficacy profile of isavuconazole as primary AFP in high-risk pediatric patients, especially in case of co-administration of immunosuppressive agents with risk of overexposure, such as ruxolitinib. Prospective pediatric trials are needed to confirm these results and to further define the role of isavuconazole as prophylactic strategy in children. However, the use of a broad-spectrum mold-active agent, such as isavuconazole for primary prophylaxis, requires careful consideration, considering possible antifungal selective pressure, potentially favoring breakthrough infections caused by intrinsically resistant or less susceptible fungi, local fungal epidemiology modification, and increase in drug-related costs.
